# The Mechanism and Function of Glia in Parkinson's Disease

**DOI:** 10.3389/fncel.2022.903469

**Published:** 2022-05-26

**Authors:** Xinguo Zhang, Ruiqi Zhang, Maher Un Nisa Awan, Jie Bai

**Affiliations:** Laboratory of Molecular Neurobiology, Medical School, Kunming University of Science and Technology, Kunming, China

**Keywords:** Parkinson's disease, microglia, astrocytes, oligodendrocytes, alpha-synuclein

## Abstract

Parkinson's disease (PD) is a neurodegenerative disease that primarily affects elderly people. The mechanism on onset and progression of PD is unknown. Currently, there are no effective treatment strategies for PD. PD is thought to be the loss of midbrain dopaminergic neurons, but it has recently been discovered that glia also affects brain tissue homeostasis, defense, and repair in PD. The neurodegenerative process is linked to both losses of glial supportive-defensive functions and toxic gain of glial functions. In this article, we reviewed the roles of microglia, astrocytes, and oligodendrocytes in the development of PD, as well as the potential use of glia-related medications in PD treatment.

## Introduction

Parkinson's disease (PD) is a common neurodegenerative disease characterized by motor and non-motor symptoms. The motor symptoms include bradykinesia, muscle stiffness, static tremor, dysfunction of postural, and gait. Non-motor symptoms consist of olfactory disability, cognitive damage, mental disorders, sleep disorders, autonomic dysfunction, soreness, and fatigue (Agarwal et al., 2020). The features of PD are the degeneration and loss of dopaminergic neurons in the midbrain, which results in the inability of downstream neural circuits and the striatum receiving adequate levels of dopamine, and induces movement symptoms. The pathology hallmark of PD is abnormal aggregation of alpha-synuclein (α-syn) protein, which is known as a major component in Lewy bodies and Lewy neurites (Agarwal and Muqit, 2021). The etiopathogenesis of PD is complicated, such as oxidative stress, inflammation, and autophagy (Barodia et al., 2019; Baumeister et al., 2019; Bido et al., 2021).

## Glia

In the central nervous system (CNS), neuroglial cells are mainly divided into microglia, astrocytes, and oligodendrocytes, which play roles in regulating inflammation, metabolism, regeneration, and myelination of neurons. However, these cells do not act alone. Recent studies demonstrate a new role for the interaction between microglia and astrocytes in the homeostasis of CNS (di Domenico et al., 2019). Moreover, microglia also acts on regulating myelination (Dzamko et al., 2017).

The microglia possess an immune function and secrete pro-inflammatory cytokines. According to the functions of microglia in the neuroinflammation, they can be categorized into two groups: poisonous to nerves (M1-phenotype microglia) and protective to nerves (M2-phenotype microglia) (Biondetti et al., 2021; Chen et al., 2021). Activated microglia secrete interleukin-1α (IL-1α), tumor necrosis-α (TNF-α), and C1q, the first subcomponent of the C1 complex, which induce astroglia into A1s (Cheng et al., 2021). In CNS lesions, infection and pathologic changes caused by activated microglia. Activated microglia operate in the inflammatory processes, interact with other cells, and affect each other (Choi et al., 2020).

Astrocyte (A1-phenotype astrocytes) also plays a critical role in producing pro-inflammatory cytokines and activating immune cells (Cichorek et al., 2021). It also plays a variety of roles in the CNS, such as regulating glucose metabolism, reuptaking glutamate, growth of synapses, and maintaining homeostasis of the blood–brain barrier (Colombo and Farina, 2016). It has been reported that astrocytes can be converted into neuronal cells (Damisah et al., 2020).

Oligodendrocytes are a type of glial cells in the CNS, which generate myelin to wrap axons. Myelin ensures the efficient propagation of action potentials along axons.

## Glia and Parkinson's Disease

Parkinson's disease is a multifactorial neurodegenerative disease in which glial cells are involved in various aspects of the disease. The dysfunctions of neurons and neural circuits in the nervous system are involved in PD. However, non-neuronal elements, like glial cells, also play a crucial role in the nervous system development, function, and plasticity (Errea and Rodriguez-Oroz, 2021). SemraSmajić et al. reported that specific neuronal cell clusters and “pan-glial” activation are involved in the pathology of the movement disorder based on single-nuclei RNA sequencing data from the idiopathic PD midbrain (Ettle et al., 2016).

Glial cells play a variety of roles in the progression of PD. Microglia and astrocytes play dual roles based on various microenvironments and cell subtypes, and produce different responses and perform different functions (Fang et al., 2021). Molecules related to familial forms of PD, α-syn (SNCA), parkin (PARK2), DJ-1 (PARK7), and ATPase 13A2 (ATP13A2 gene) work together with microglial and astrocyte activation (Filippini et al., 2021; Fu et al., 2021). Oligodendrocytes are associated with demyelination in PD (Fujita et al., 2018).

### Microglia and PD

Microglia are involved in the pathological process of neuroinflammation and neurodegenerative diseases, including PD. The α-syn aggregates in neurons which secrete exosomes carrying α-syn out of cells and activates an inflammatory response in microglia and astrocytes (Glass et al., 2010). These exosomes target microglia preferentially (Gordon et al., 2018). Fyn kinase is a protein tyrosine kinase known to regulate proinflammatory effects in T cells and other immune cells and contributes to astrocytic migration and the differentiation of oligodendrocytes in CNS. Fyn plays the role in activating the NLR family pyrin domain containing 3 (NLRP3) inflammasome in any cell types. The FYN gene is identified as a novel PD risk locus in a genome-wide association study (GWAS). Fyn conjuncts with the class B scavenger receptor CD36, then facilitates α-syn importing into microglia (Guo et al., 2020). Furthermore, microglia Kv1.3, a voltage-gated potassium channel, is transcriptionally upregulated and post-translationally modified by Fyn. Whereas, small-molecule PAP-1, an inhibitor of Kv1.3 can inhibit neuroinflammation regulated by Kv1.3 in neurodegeneration (Hanslik et al., 2021). The human microglial transcriptome study showed that microglial expression of P2Y_12_R is associated with PD (Hentrich et al., 2020). P2Y_12_R regulates ras homolog family member (Rho)-associated coiled coil-containing protein kinase (ROCK) and p38 MAPK activity and controls cytokine production. This receptor plays a dual role in PD: P2Y1_2_Rs are necessary for the initiation of protective inflammatory response, then maintain the activation of microglia and stimulate the pro-inflammatory cytokine response at later stages of neurodegeneration (Hughes and Appel, 2020). Triggering receptors expressed on myeloid cells 2 (TREM2), phosphoinositide-specific phospholipase C (PLC) γ2, and protein kinase C (PKC) promote the activation of reparative/regenerative microglial subtypes which are beneficial for neurodegenerative diseases (Hughes et al., 2019).

The Toll-like receptors (TLRs) signaling pathway is the primary signaling pathway that mediates the inflammatory response. The α-syn sensitizes the TLR4-dependent inflammatory response (Kon et al., 2019) and decreases the microglial glucocorticoid receptors (GR) expression. In the absence of GR in microglia, TLR9 translocation to endolysosomes is enhanced and the cleavage of TLR9 is also promoted, which leads to pro-inflammatory gene expression (Kwon and Koh, 2020). Then microglia secrete cytokines and promote the death of neurons. Furthermore, the neuronal expression of TLR2 is significantly increased by α-syn, and the activated TLR2 leads to neuroinflammatory response, the production of reactive oxygen species (ROS), the secretion of inflammatory cytokines, and the microglial-activating chemokines (Lacagnina et al., 2017).

The NLRP3 inflammasome is related to neuroinflammation activation in microglia. NLRP3 inflammasome then leads to Cleaved caspase-1 and the inflammasome adaptor protein apoptosis-associated speck-like protein containing a C-terminal caspase recruitment domain (ASC) elevation (Lai et al., 2021), and the release of interleukin-1β (IL-1β). The α-syn can mediate the generation of mitochondrial ROS leading to the activation of the NLRP3 inflammasome (Guo et al., 2020). 1-methyl-4-phenyl-1,2,3,6-tetrahydropyridine (MPTP), used for making mouse model of PD, induces mitochondrial ROS in neural cells, promotes the robust assembly, and activates the NLRP3 inflammasome (Lee et al., 2019).

The β-arrestin1 (ARRB1) and β-arrestin2 (ARRB2) play the opposite roles in microglia-mediated inflammation, as well as in the pathogenesis of PD (Liddelow and Barres, 2017). ARRB1 aggravates, whereas ARRB2 ameliorates, the pathological features of PD. The p65, a component of the NF-κB pathway can interact with these two ARRBs which lead to adverse effects on inflammation by activating signal transducers and activators of transcription 1 (STAT1) and NF-κB pathways. These two molecules exert different functions in regulating the expression of nitrogen permease regulator-like 3 (Nprl3), which conversely mediates the functions of both ARRBs in microglial inflammatory responses. Fyn kinase mediates NF-κB–p65 nuclear translocation through PKC-δ (PKCδ) pathway and promotes the initiation of inflammasome (Guo et al., 2020).

Leucine-rich repeat kinase 2 (LRRK2) plays a critical role in the microglial inflammatory response mediated by TLR 2 (Liddelow et al., 2017). The α-syn induces the phosphorylation and activity of LRRK2, then LRRK2 selectively phosphorylates and also induces the nuclear factor of activated T cells, cytoplasmic 2 (NFATc2) translocation into the nucleus, then promotes a neuroinflammatory cascade. Inhibition of LRRK2 apparently decreases microglial neurotoxicity mediated by α-syn by reducing the level of TNF-α and IL-6. In microglia, LRRK2 modulates IFN-γ stimulated the production of cytokine in an NFAT-independent way (Lin et al., 2021). INF-γ increases neuronal susceptibility to immune challenges by enhancing the LRRK2 G2019S-dependent negative regulation of Protein Kinase B (also known as AKT) phosphorylation and NFAT activation. Microglia accumulating α-syn aggregation results in a reactive state with phagocytic function, excessively produces oxidative and proinflammatory molecules, and selectively recruits peripheral immune cells (Lopes et al., 2022). These peripheral immune cells secrete IFN-γ, create a vicious molecular feed cycle with microglia, and induce neurons to apoptosis.

The engulfed α-syn mediates the upregulation of E3 ubiquitin-protein ligase pellino homolog 1 (PELI1), which impairs the autophagy flux and induces the α-syn aggregation in microglia (Iring et al., 2021). After exceeding its degradation capacity, the excessive α-syn is released from microglia to the extracellular matrix, and is taken into neurons resulting in the transferring of α-syn from cell to cell (Kalia and Lang, 2015). Thus, the α-syn inhibits microglia autophagy and promotes neurodegeneration (Kierdorf and Prinz, 2013). In the meantime, the α-syn activates microglial TLR4, which induces the transcriptional upregulation of p62/SQSTM1(Sequestosome-1), selective autophagy of the adaptor protein through the NF-κB signaling pathway, then p62 facilitates the formation of α-synuclein/ubiquitin-positive complex which is degraded by synucleinphagy (a special form of autophagy), thus protecting neurons (Kim et al., 2020). The p38 MAPK *via* T-cell Transcription Factor EB (TFEB) inhibits chaperone-mediated autophagy (CMA)-mediated NLRP3 degradation which activates microglia. SB203580 is the inhibitor of p38 MAPK and alleviates movement dysfunction by preventing neurodegeneration *in vivo*. MCC950, a small-molecule NLRP3 inhibitor has the ability to abolish inflammasome activated by fibrillar α-syn in microglial cells, slow down the nigrostriatal dopaminergic degeneration, and mitigate motor deficits (Kim and Kornberg, 2021).

It has been reported that iron content increases in the substantia nigra pars compacta (SNpc) is a reason for dopaminergic striatal dysfunction and cell loss (Loria et al., 2017). Iron content in SN and GP (paleostriatum) gradually elevated in the whole process of PD (Maatouk et al., 2018). Iron deposition may be a feature of preclinical and early stage of this disease; thus deserves more attention. LRRK2 mutations linked to PD sequester Rab8a to damage lysosomes and regulate transferrin-mediated iron uptake in microglia. LRRK2 modulates iron uptake and storage in microglia after the activation of proinflammatory molecules (Maatouk et al., 2019). Lin and their colleagues found that DJ-1 regulated microglial activation in response to lipopolysaccharide (LPS) treatment. DJ-1 deficiency in microglia increases the neurotoxicity induced by LPS (Mamais et al., 2021).

**Figure 1:**
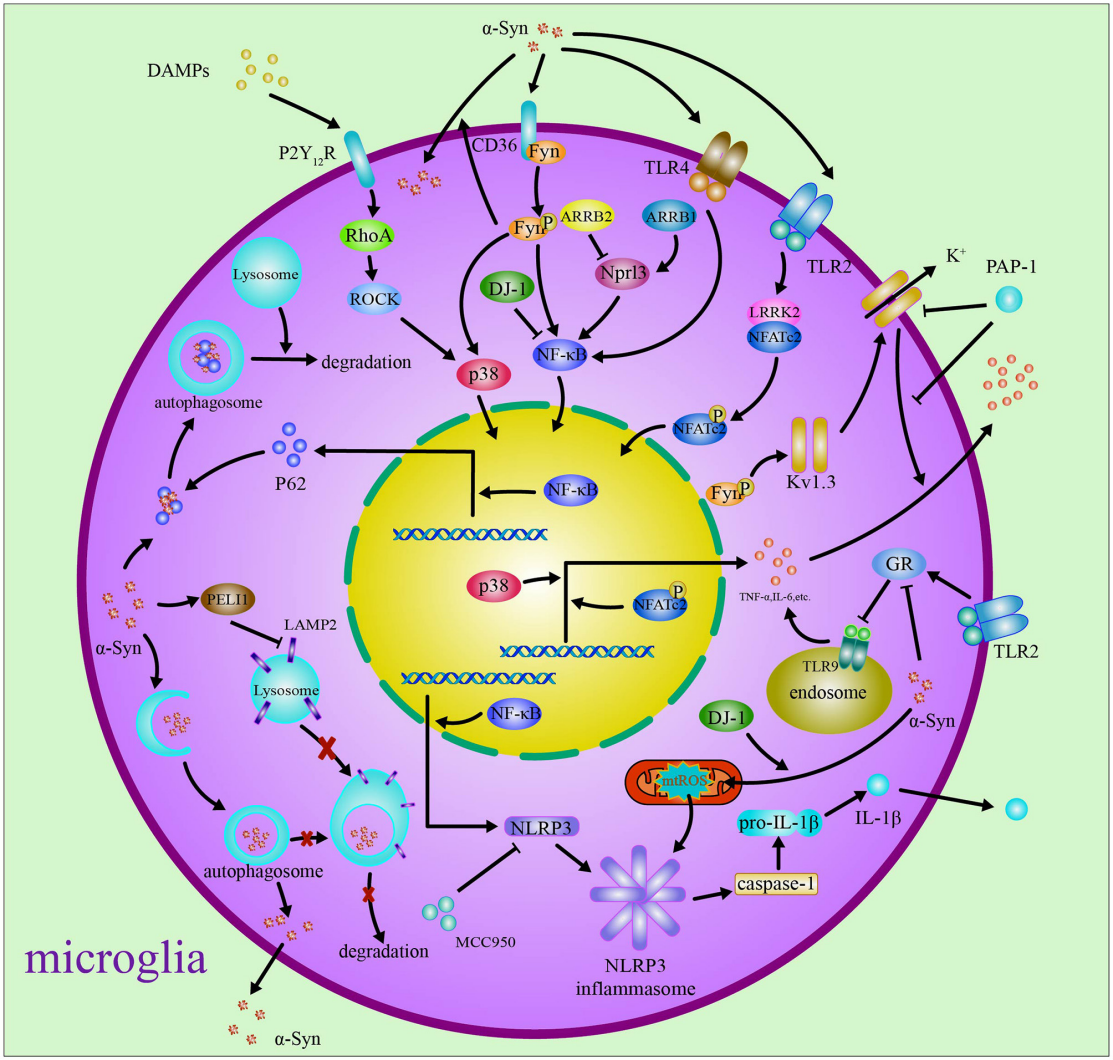
Microglia in Parkinson's disease. In Parkinson's disease, debris containing α-syn and DAMPs from neurons activates microglia through regulating Fyn, Kv1.3, and P2Y_12_R *via* ROCK and p38 MAPK pathways. The α-syn induces neuroinflammatory response through activating TLR4/TLR9/TLR2, NLRP3 inflammasome, ARRB1/ARRB2/STAT1/NF-κB/NLPR3, and LRRK2/NFAT pathways. The phagocytized α-syn impairs the autophagy through PELI1, TLR4/p62/SQSTM1, and p38/TFEB/NLRP3 pathways.

Based on the above reviews, it has been shown that the role of microglia in PD is closely related to α-syn. The α-syn activates microglia to induce neuroinflammatory response and inhibits autophagy and finally results in neurotoxicity in PD. Thus, regulation of neuroinflammatory and autophagy in microglia is the therapy strategy for PD. The roles and the related mechanism of microglia in PD are included in Figure 1.

### Astrocyte and PD

Astrocytes phagocytose and degradate α-syn fibrils *in vitro* and in *ex vivo* brain sections (Morales et al., 2017; Oksanen et al., 2019). 6-hydroxydopamine is used to establish a PD animal model by destroying dopaminergic neurons in the striatum. In the rat model of 6-hydroxydopamine, it was observed that astrocytes phagocytosed dopaminergic debris and α-syn, which suggests that astrocytes may have a beneficial role in clearing damaged cellular components in PD (Panicker et al., 2019; Panagiotakopoulou et al., 2020). Astrocytes have also been reported to prevent dopaminergic neurons from α-syn accumulation and spread (Panicker et al., 2019). However, in the later stage of PD, astrocytes may be induced into the A1 reactive state, which is highly cytotoxic to neurons and oligodendrocytes rather than phagocytic (Cheng et al., 2021).

Connexin 30 (Cx30) is an astrocytic gap junction protein. A2 astrocytes reduce dopamine (DA) neuron loss in Cx30 knockout mice, which indicates that Cx30 plays a critical role in PD (Picca et al., 2021). The reduction of GR increases Connexin 43 (Cx43) hemichannel activity and elevates intracellular calcium levels in astrocytes, which induces the elevation of astrocyte specific inflammation-associated transcripts, including intercellular cell adhesion molecule-1 (ICAM-1), TNF-α, and IL-1β, as well as the excessive production of microglia (Qian et al., 2020).

Compared with neurons, astrocytes also exhibit higher internalization of α-syn and lysosomal degradation rate (Reyes et al., 2019). This internalization of α-syn is blocked by the protein clusterin (Rivetti di Val Cervo et al., 2017). Astrocytes are able to efficiently degrade fibrillar α-syn through lysosomal degradation, which decreases the accumulation of α-syn in neurons and protects them in this way (Morales et al., 2017). During PD pathogenesis, dysfunctional chaperone-mediated autophagy (CMA) and impaired macroautophagy lead to accumulation of α-syn in astrocytes (Panicker et al., 2019). Then astrocytes secrete majority of their internalized protein aggregates, most of which are engulfed and cleared by microglia (Rostami et al., 2020). Thus, the clearance of α-syn is mainly undertaken by microglia. Furthermore, α-syn fibril oligomers induce astrocytes to release Ca^2+^-dependent glutamate, which then activates glutamate receptors like extrasynaptic N-methyl-D-aspartic acid receptor (NMDA) receptors (eNMDARs). Thus, NitroSynapsin (an NMDAR antagonist) can reverse eNMDAR-mediated synaptic loss induced by oligomeric α-syn (Rostami et al., 2021).

The α-syn is able to activate both non-specific immune responses and specific immune responses (Sarkar et al., 2020). It induces astrocytes to express high levels of MHC-II to present α-syn (Siokas et al., 2022). Then T cells infiltrate into the CNS, including Th1/Th2 which produce Th1/Th2 cytokines, CD4+ and CD8+ T cells which secrete IFN-γ. This kind of reaction aggravates the loss of dopaminergic cells.

Just like in microglia, NLRP inflammasome plays an important role in astrocytes. Dopamine D2 receptor (Drd2) inhibits the activation of NLRP3 by regulating β-arrestin2 interaction with NLRP3 and interferes with the inflammasome assembly. In this way, Drd2 decreases caspase-1 expression and reduces IL-1β release (Smajic et al., 2021).

Experiments have shown that astrocytes are able to convert into neurons, and such strategies have considerable therapeutic potential in the treatment of PD. It has been studied that the transcription factors, ASCL1, LMX1A, NEUROD1, and NeAL218 (microRNA miR218) reprogram astrocytes into induced dopamine neurons (iDANs), and improve the efficiency of reprogramed astrocytes by promoting the chromatin remodeling and activating the transforming growth factor-β (TGF-β), Shh, and Wnt signaling pathways (Spaas et al., 2021). Phosphatase and tensin homolog deleted on chromosome ten (PTEN)-induced putative kinase 1 (PINK1)-dependent ubiquitin phosphorylation is predominantly in astrocytes, which suggests that PINK1 is related to astrocyte dysfunction in PD (Stok and Ashkenazi, 2020). Another study showed that depleting the polypyrimidine tract binding protein (PTB) converted astrocytes into functional neurons (Tremblay et al., 2019). Interestingly, astrocytes from different brain regions convert into different neuronal subtypes. Midbrain astrocytes convert into dopaminergic neurons whose axons reconstruct the nigrostriatal circuit, restore dopamine levels, and rescue the motor deficits in PD.

**Figure 2:**
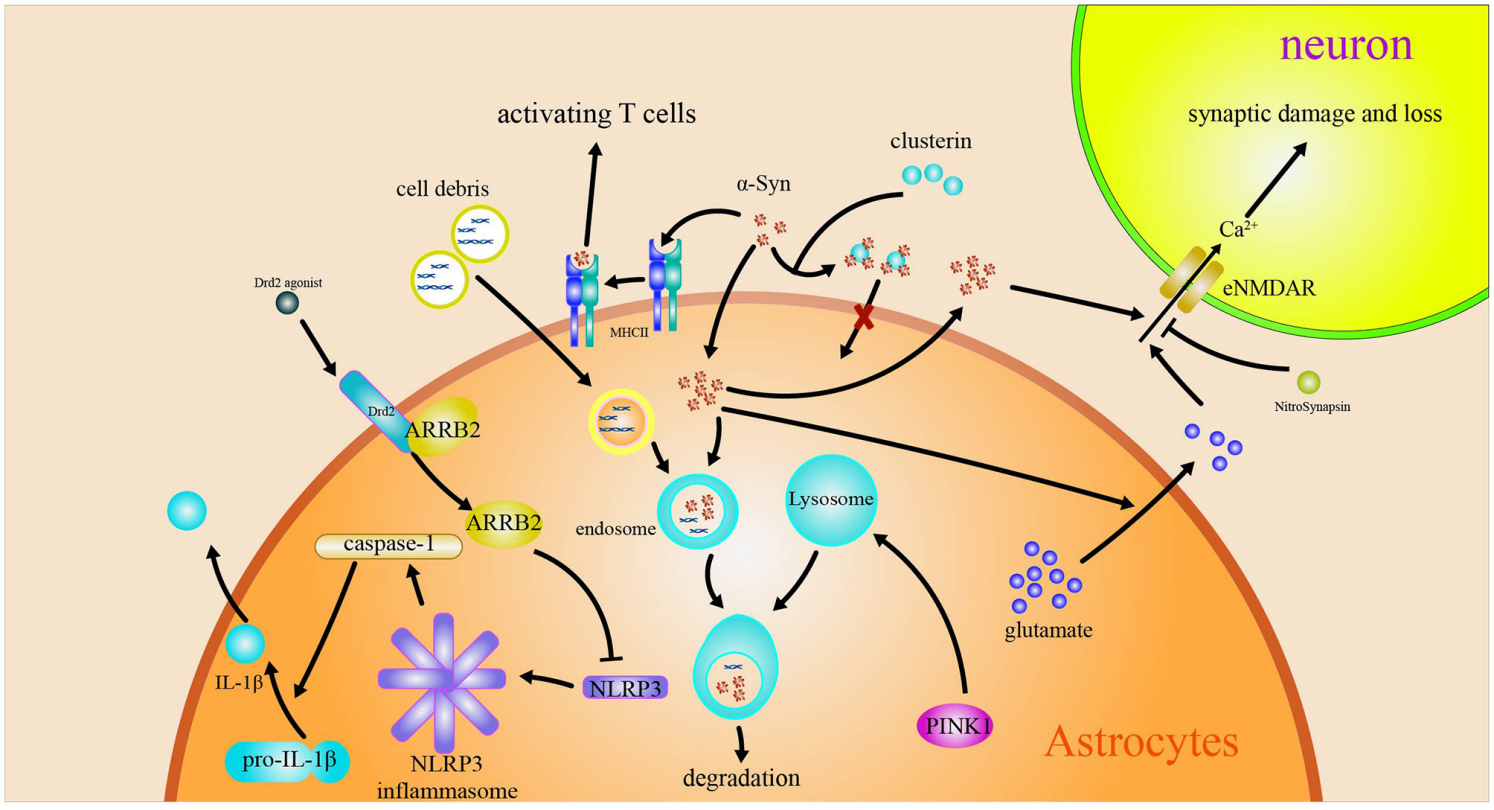
Astrocytes in Parkinson's disease. The engulfed α-syn has a variety of effects on astrocytes. The α-syn induces MHC-II expression in astrocytes and activates T cells. The α-syn induces the release Ca2+-dependent glutamate and activates eNMDARs to accelerate the injury and loss of synapsis. Drd2 inhibits the activation of NLRP3 by regulating β-arrestin2.

**Figure 3:**
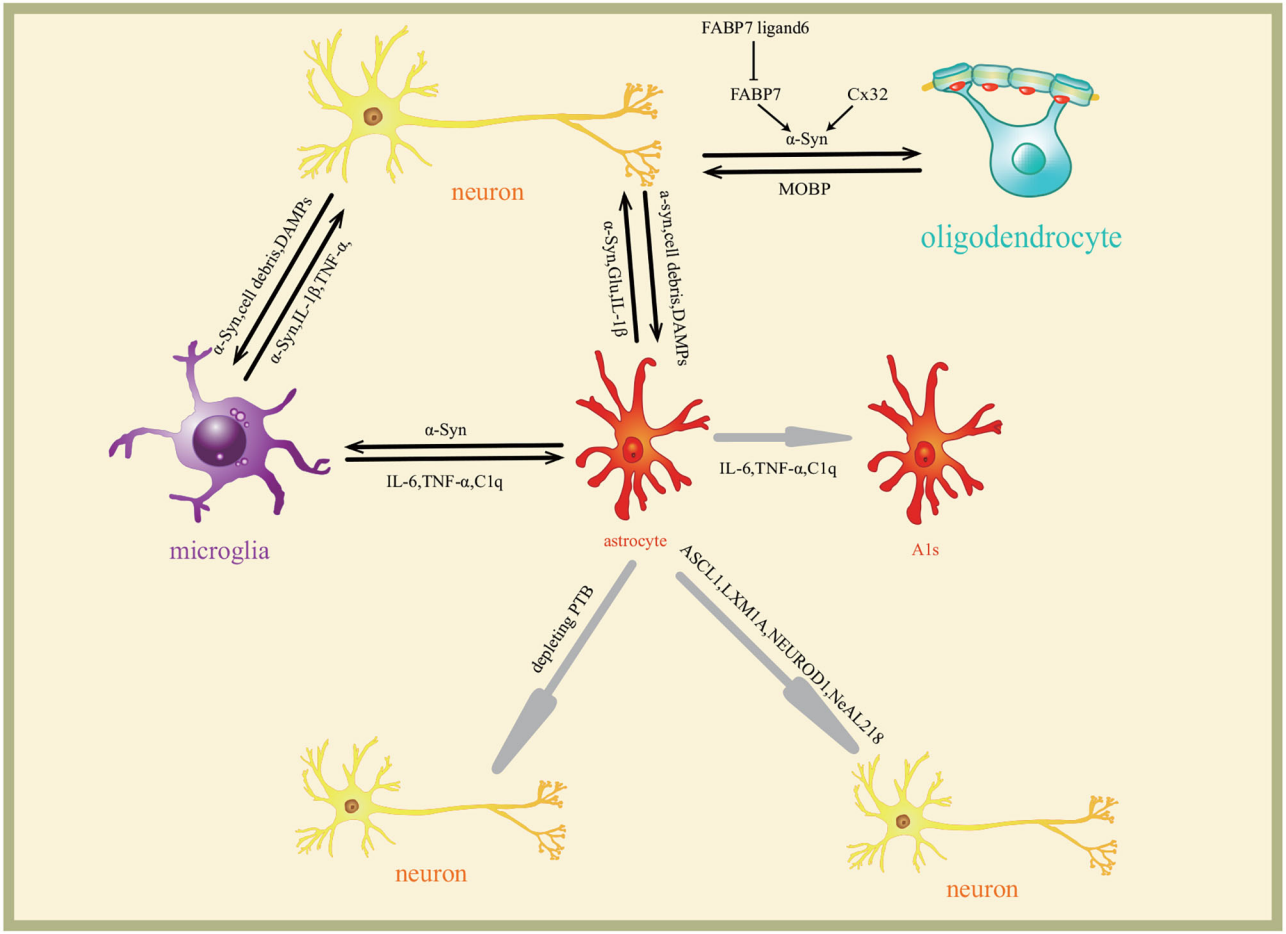
Interaction of glia and neurons in Parkinson's disease. Neurons secrete α-syn to extracellular matrix which induces glia reaction differently. The molecules are transmitted among neurons and glia to form a complex regulating network. Astrocytes are induced to A1s by microglia through inflammatory factors IL-6, TNF-α, and C1q. Astrocytes are converted into functional neurons by ASCL1, LMX1A, NEUROD1, and NeAL218 transcription programs. Cx32 and FABP7 regulate the oligodendrocytes survival.

The above-mentioned reviews suggest that the roles of astrocytes in PD are closely related to the inflammation, macroautophagy impairments, and MHC-II regulating Th1/Th2 cytokines production induced by α-syn. Astrocytes are converted into functional neurons. The roles of and the related mechanism of astrocytes in PD are included in Figure 2. The pathway on the astrocytes converted into neurons is shown in Figure 3.

### Oligodendrocytes and PD

Oligodendrocytes are differentiated from oligodendrocyte precursor cells (OPCs). OPCs are composed of 5% of the resident parenchymal central nervous system glial cells. More and more evidence shows that OPC dysfunction and the lack of OPC differentiation participate in the progression of neurodegenerative disorders, such as PD (Trudler et al., 2021). White matter myelin profiles are linked to clinical subtypes of PD (Tsunemi et al., 2020).

The progression in oligodendrocytes has been detected in multiple system atrophy (MSA), a rare and atypical PD (Tu et al., 2021). An analysis sequence of about 17,000 nuclei from matched SN samples found that dopaminergic neuron-specific gene expression makes up the crucial genetic risk for PD, such as protein folding and ubiquitination pathways, mitochondrial functioning, and a distinct cell type associated with PD and oligodendrocyte-specific gene expression (Vizziello et al., 2021).

Myelin-associated oligodendrocyte basic protein (MOBP) polymorphism is one of the risk factors for PD (Williams et al., 2021). Furthermore, the expression of myelin-associated gene is increased in the frontal cortex of α-syn overexpressing rats and PD patients (Xia et al., 2019). Myelin loss is associated with α-syn accumulation in oligodendrocytes (Tu et al., 2021). In PD and dementia with Lewy bodies, MOBP is found in LBs originated from the brainstem, cingulate cortex, and sympathetic ganglia. However, lots of evidence shows that MOBP doesn't occur in other inclusions of neurons and glia in other neurodegenerative diseases, such as multiple system atrophy, Alzheimer's disease, and Pick's disease. It has been reported that MOBP is upregulated in neurotoxic conditions, indicating that accumulation of MOBP in LBs may play a role in protecting cells in LB disease (Xia et al., 2021).

The previous studies show that the gap junction protein connexin-32 (Cx32) is crucial for the uptake of α-syn in neurons and oligodendrocytes and targets Cx32 to block α-syn uptake (Yellajoshyula et al., 2022). Benztropine restores the α-syn-induced myelination deficit of oligodendrocytes.

Extracellular vesicles secreted from the PD brain to peripheral blood contain a higher level of α-syn. Compared to MSA patients, the peripheral blood of PD patients contains a higher concentration of oligodendrocyte-derived enriched microvesicles (OEMVs), whereas the average concentration of α-syn in each OEMVs shows no significant difference between these two groups. The mechanism is probably that α-syn interferes with the interaction between vesicle-associated membrane protein 2 (VAMP2) and syntaxin 4, causing the dysfunction of the soluble N-ethylmaleimide-sensitive factor attachment protein receptor (SNARE) complex. This study shows the potential method to discern PD with MSA (Yu et al., 2020).

Activated phospholipase A2 (PLA2) triggers fatty acid-binding protein 7 (FABP7) forming a complex with endogenous α-syn (Zhu et al., 2018). The oligomer induces cell death of both oligodendrocytes and OPCs. This oligomerization and aggregation of FABP7 with α-syn are significantly inhibited by FABP7 ligand 6, so FABP7 ligand 6 prevents oligodendrocytes and OPCs from cell death. The role and the mechanism of oligodendrocytes in PD are shown in the part of oligodendrocytes in Figure 3.

Although the role of oligodendrocytes in PD has not been studied well, oligodendrocytes play active roles in PD due to its important role in myelin formation. Indeed, the α-syn accumulation in oligodendrocytes is related to myelin loss. More studies on the relationship between PD and oligodendrocytes are needed.

## Conclusion and Discussion

Although α-syn induces the response of various cells in CNS, recent studies also suggest that microglia and astrocytes play a minor role in the development of PD (Vizziello et al., 2021), the neurons themselves are more related to PD. The microglia and astrocytes play the role in regulating the neuroinflammatory response induced by α-syn. The astrocytes secrete α-syn aggregates, most of which are engulfed and cleared by microglia. The astrocytes are also involved in oligodendrocytes. The astrocytes are converted into functional neurons. The roles and the interaction among microglia and astrocytes, oligodendrocytes, and neurons in PD are included in Figure 3.

Understanding what happened in the neurons prior to the generation of α-syn, and transferring in multi cells is an attractive research direction on PD. The research about the initiation of PD will help to understand the molecular mechanism of PD accurately and has significance in clinically preventing and treating it. However, because PD symptoms are not significant during this period, research may be difficult. Thus, paying attention to glia roles in the CNS is important for preventing PD early.

## Author Contributions

XZ wrote the review. RZ participated in the discussion. MN and JB revised the review. All authors contributed to the article and approved the submitted version.

## Funding

This work was supported by the National Natural Science Foundation of China (Nos. U2002220 and 81660222); the Yunling Scholar (No. 1097821401); and the key lab for oxidative stress damage and defense in the University of Yunnan Province (2018).

## Conflict of Interest

The authors declare that the research was conducted in the absence of any commercial or financial relationships that could be construed as a potential conflict of interest.

## Publisher's Note

All claims expressed in this article are solely those of the authors and do not necessarily represent those of their affiliated organizations, or those of the publisher, the editors and the reviewers. Any product that may be evaluated in this article, or claim that may be made by its manufacturer, is not guaranteed or endorsed by the publisher.
